# Hospital Festivals and Meetings

**Published:** 1895-03-09

**Authors:** 


### HOSPITAL FESTIVALS AND MEETINGS.

### MIDDLESEX HOSPITAL.

A quarterly general court of the governors of the Middlesex Hospital was held on February 28th, 1895, when the annual report for 1894 was adopted. The chair was taken by Mr. A. PEARCE GOULD, M.S., and amongst others present were: Mr. Francis Hoare (the treasurer), Mr. Bell Sedgwick, J.P. (deputy chairman), Mr. G. B. Hudson, M.P., Mr. Elliott Lees, M.P., Colonel Needham, Mr. Griffith Jarrett, Mr. Kegan Paul, the Rev. Canon Acheson, and Mr. Leopold Hudson, F.R.C.S.

The SECRETARY-SUPERINTENDENT (Mr. Clare Melhardo) read the report for 1894, and in the absence of Sir Ralph Thompson (chairman of the weekly board), through illness, its adoption was moved by Mr. KEGAN PAUL, and seconded by Mr. BELL SEDGWICK.

Mr. Treasurer HOARE reported that £5 10s. 1d. was collected from the alms boxes within the hospital during the past quarter, and £74 from the boxes in the workshops and business establishments in the neighbourhood.

The Marquis of Breadalbane and Lord Sandhurst were elected vice-presidents; and Mr. Francis Hoare, Mr. G. J. Marjoribanks, and the Hon. Walter Rothschild were re elected treasurers for the ensuing year. Ordinary business was then transacted, and the meeting closed with a cordial vote of thanks to the chairman.

The report stated that it was not possible, as usual, to make a detailed comparison of statistics of income, expenditure, and cases, &c., of the past year with that of the one immediately preceding, because in 1893 the hospital was closed for nearly three months, and the number of patients admitted was far below the normal in consequence. There had been a certain falling off in ordinary income, which was partly to be attributed to the large contributions resulting from the festival dinner, which brought in a sum of £8,452 5s. Annual subscriptions were slightly in excess of those for 1893. The award from the Metropolitan Sunday Fund was the largest save one ever received. The thirty-second annual entertainment given by the employés of Messrs. Marshall and Snelgrove on behalf of the hospital brought in a contribution of £48 10s. 8d. A new and more liberal scale of diet for the nursing staff had been introduced, and the number of hospital servants increased. The new convalescent home at Clacton-on-Sea was reported to be progressing, the roofing-in was to be accomplished as soon as the weather admitted. The reconstruction of the operating theatre had also been carried out during the past year; it provided accommodation for 100 students, and was fully equipped with every necessary appliance. Among the various changes of the past year the report specially commented upon the loss of Lord Sandhurst's services as chairman of the board, in consequence of his appointment as Governor of Bombay (Sir Ralph Thompson, K.C.B., having been elected in his stead), and upon the great loss sustained by the hospital in the recent death of the senior surgeon, Mr. John Whitaker Hulke, F.R.S., who for a period of 33 years "performed his duties . . . with a zeal only equalled by his ability, and endeared himself to all who came in contact with him by his invariable kindness and courtesy." A very satisfactory account was given of the work of the nursing department.

### CANCER HOSPITAL, BROMPTON.

The annual general meeting of this institution was held on Wednesday, February 27th. Sir George S. Measom presided. The committee reported that the number of patients treated during the past year was greater than ever before; 2,500 new patients had been under treatment, 787 in-patients and 1,513 out-patients. The help rendered to poor patients, when discharged, by the Samaritan Fund had been invaluable, both in sending patients to convalescent homes, or in certain cases in providing pecuniary aid.

### ROYAL FREE HOSPITAL.

The sixty-seventh Annual Court of the Governors of the Royal Free Hospital was held on Wednesday, February 27. The chair was taken by Sir Julian Goldsmid, M.P., one of the vice-presidents of the hospital. There was a good attendance of governors, amongst whom were Sir Gainsford Bruce, General Gordon Pritchard, C.B., Colonel Montefiore, Mrs. Garrett Anderson, M.D., Dr. T. Crawford Hayes; but influenza was responsible for the non-appearance of many who would otherwise have been present. The report for 1894 was read by the secretary, Mr. Conrad W. Thies. In moving its adoption, the Chairman stated that the new front building completed the work of reconstruction commenced forty years since, and provided greatly improved accommodation for casualty patients, dispensary, resident medical officers and servants, also much needed isolation wards; and that many other important and necessary improvements have been carried out in various parts of the hospital. The total cost of the works had been about £30,000, towards which the sum of £24,000 had been raised, leaving a deficit of about £6,000. He appealed to the public to help the committee by providing the sum still needed, so that the new buildings might be opened free from debt. The medical reports showed that the number of patients treated during the past year was 30,337, as compared with 28,274 in the preceding year. The total ordinary receipts for 1894 (exclusive of legacies), had been £5,255 13s. 9d., against expenditure amounting to £9,758 7s. 6d., the deficiency having been met partly by the legacies received and partly by encroaching upon the small reserve funds. The chairman strongly urged the claims of the charity upon the benevolent public, and stated that the small income derived from annual subscriptions was most inadequate, and he trusted that this would be augmented in the future. The new front buildings and laundry were open for inspection during the afternoon—Among the necessary improvements which have been accomplished in the hospital may be enumerated the entire repainting of external and internal wood and iron work; extensive alterations to ward lavatories and bath-rooms, thus ensuring better sanitation; and additional accommodation for the students. The floors of some of the wards are also to be relaid with oak. Lifts, and the introduction of the electric light, are improvements still to come; but for want of funds these are postponed for the present. During the past year the regulations relating to the nursing staff have undergone certain changes—the period of training has been extended from three to four years, an increase has been made in the salaries of the sisters in charge of wards and their summer holidays extended from three to four weeks, while probationers will in future be allowed three weeks' leave of absence each year.

### NATIONAL ORTHOPÆDIC HOSPITAL.

The annual meeting of the National Orthopædic Hospital was held on Thursday, February 28th. The chair was taken by Mr. J. R. Cooper, chairman of the committee of management. The attendance of governors was very small, the non-attendance of many being due to the all-prevailing influenza. The annual report was in every way a satisfactory one, and recorded an increase of "fully 20 per cent." in the ordinary income for the past year, the figures showing a total of £2,116 13s. 5d. (including subscriptions, donations, and patients' payments) for 1864, as against a total of £1,710 0s. 4d. for 1893. The increase was most marked in the case of donations. A corresponding increase was recorded in the number of patients attending the hospital and consequently in the expenditure. 216 in-patients and 1,259 out-patients were treated during the year, and at its close, all the beds having been continuously occupied, some 30 patients were awaiting admission. The committee announced that the treasurership of the hospital had been accepted by Sir Horace Farquhar, Bart., on the resignation of Mr. Francis Hoare. It had been decided, on the suggestion of the medical staff, to open the hospital to out-patients on one evening in the week, for the benefit of those who are employed during the day. Special anxiety was felt with regard to clearing off the debt to the bankers contracted during the building of the new portion of the hospital in 1892. In commenting upon the report, Mr. Cooper said he thought the record of work was excellent. During the last ten years the hospital had doubled both its income and its patients, and it was satisfactory that there were so many new subscriptions, the backbone of a hospital's income. He was glad to note how popular the free bed letters were becoming, the number issued having steadily grown each year since the idea was introduced. Mr. Cooper alluded to the loss sustained by the hospital in the death of the late secretary, Mr. William Tresidder, the work now being carried on by his brother. The report having been adopted, cordial votes of thanks were given to the medical staff, secretary, and auditors, and specially appreciative mention was made by Mr. Muirhead Little of the untiring and helpful labours of the matron.

